# Twenty-four-hour movement guidelines during adolescence and its association with obesity at adulthood: results from a nationally representative study

**DOI:** 10.1007/s00431-022-04760-w

**Published:** 2022-12-21

**Authors:** Antonio García-Hermoso, Yasmin Ezzatvar, Alicia M. Alonso-Martinez, Robinson Ramírez-Vélez, Mikel Izquierdo, José Francisco López-Gil

**Affiliations:** 1 Navarrabiomed, Hospital Universitario de Navarra (HUN), Universidad Pública de Navarra (UPNA), IdiSNA, Pamplona, Spain; 2 Department of Nursing, Universitat de València, Valencia, Spain; 3 Department of Health Sciences, Public University of Navarra, CIBER of Frailty and Healthy Aging (CIBERFES), Instituto de Salud Carlos III, Pamplona, Navarra Spain; 4 Health and Social Research Center, Universidad de Castilla-La Mancha, Cuenca, Spain

**Keywords:** Physical activity, Screen time, Sleep duration, Body mass index, Waist circumference

## Abstract

To determine the association between adherence to the 24-h movement guidelines during adolescence with obesity at adulthood 14 years later in a nationally representative cohort. We analyzed data from 6984 individuals who participated in Waves I (1994–1995) and IV (2008–2009) of the National Longitudinal Study of Adolescent Health (Add Health) in the USA. Obesity was defined by the International Obesity Task Force cut-off points at Wave I and adult cut-points at Wave IV (body mass index [BMI]≥30 kg/m2 and waist circumference [WC]≥102 cm in male and 88 cm in female). Physical activity, screen time and sleep duration were self-reported. Adolescents who met screen time recommendation alone (β = −1.62 cm, 95%CI −2.68 cm to −0.56), jointly with physical activity (β = −2.25 cm, 95%CI −3.75 cm to −0.75 cm), and those who met all three recommendations (β = −1.92 cm, 95%CI −3.81 cm to −0.02 cm) obtained lower WC at Wave IV than those who did not meet any of these recommendations. Our results also show that meeting with screen time recommendations (IRR [incidence rate ratio] = 0.84, 95%CI 0.76 to 0.92) separately and jointly with physical activity recommendations (IRR = 0.86, 95%CI 0.67 to 0.97) during adolescence is associated with lower risk of abdominal obesity at adulthood. In addition, adolescents who met all 24-h movement recommendations had lower risk of abdominal obesity later in life (IRR = 0.76, 95%CI 0.60 to 0.97).

*Conclusion*: Promoting the adherence to the 24-h movement guidelines from adolescence, especially physical activity and screen time, seems to be related with lower risk of abdominal obesity later in life, but not for BMI.

**Table:** 

**What is Known:** *• Some studies have shown a relationship between adherence to 24-h movement guidelines and adiposity or obesity markers in youth. However, most of these studies have a cross-sectional design or a short follow-up.*	
**What is New:** *• This is the first study which determined the association between adherence to the 24-h movement guidelines during adolescence with obesity at adulthood 14 years later in a nationally representative US cohort.* *• Meeting the 24-h movement guidelines from adolescence seems to be related with lower risk of abdominal obesity later in life, but not for body mass index.*

**What is Known:**

*• Some studies have shown a relationship between adherence to 24-h movement guidelines and adiposity or obesity markers in youth. However, most of these studies have a cross-sectional design or a short follow-up.*

**What is New:**

*• This is the first study which determined the association between adherence to the 24-h movement guidelines during adolescence with obesity at adulthood 14 years later in a nationally representative US cohort.*

*• Meeting the 24-h movement guidelines from adolescence seems to be related with lower risk of abdominal obesity later in life, but not for body mass index.*

## Introduction

The prevalence of obesity is rapidly increasing worldwide, which may portend undesirable adverse health effects [1], such as an increase in the incidence of cardiovascular disease risk factors and a greater risk for cardiovascular morbidity and mortality in adulthood [2, 3]. As the burden of obesity and related noncommunicable diseases continues to rise in USA [4], there is a need for research on modifiable risk factors in adolescence that could be integrated into intervention strategies.

Existing evidence indicates that the composition of movement behaviors (e.g., physical activity, sedentary behavior, and sleep duration) are time-dependent, eliciting distinct biological processes that interact throughout a 24-h period [5]. Meeting current 24-h movement guidelines is linked to several health outcomes in young population [6] and later during adulthood [7]. However, from adolescence to adulthood, the time spent in some of these behaviors has shown to be reduced (e.g., physical activity [8], sleep duration [9]), and increased in others (e.g., sedentary behavior [10]). Adolescence is characterized by fast physical growth and modifications in body composition generated by the hormonal fluctuations related to puberty [11]. Further, the health-related behaviors that influence on excess weight in adulthood frequently begin or are strengthened during adolescence [12]. Thus, the adherence to healthy behaviors during adolescence can offer both short and long-term benefits on health [13].

Current 24-h movement guidelines recommend that a healthy 24-h day in adolescents should include at least 60 min of moderate-to-vigorous physical activity (MVPA), no more than 2 h of screen time, and 8–10 h of sleep [5]. However, a recent meta-analysis reported that only 2.68% of adolescents from 23 countries met all these recommendations for MVPA, screen time, and sleep [14]. The healthy behaviors that compound the 24-h movement guidelines are modifiable and have been independently linked to body composition (e. g., physical activity [15], sedentary behavior [15], and sleep duration [16]). More favorable movement patterns have been associated with reduced adiposity or obesity in cross-sectional studies [17–21]. The few existing prospective studies also reported that not meeting the guidelines in childhood is associated with higher adiposity at follow-up [22–24]. For example, Leppänen et al. [24] showed that meeting most of the guidelines was longitudinally associated with lower abdominal obesity two years later. Chemtob et al. [22] also suggested that not meeting the guidelines in childhood is associated with higher adiposity 2 and 7 years later. This finding is consistent with that of Micklesfield et al. [23] who suggested that patterns of adolescent physical activity, sedentary behavior and sleep are related to young-adult body composition in urban South Africa. Nevertheless, none of the previous studies has reported this association between 24-h movement behaviors during adolescence and obesity at mid-adulthood. Therefore, the aim of our study was to determine the association between adherence to the 24-h movement guidelines during adolescence with obesity at adulthood in a nationally representative US cohort.

## Material and methods

### Population sample and study design

**Fig. 1:**
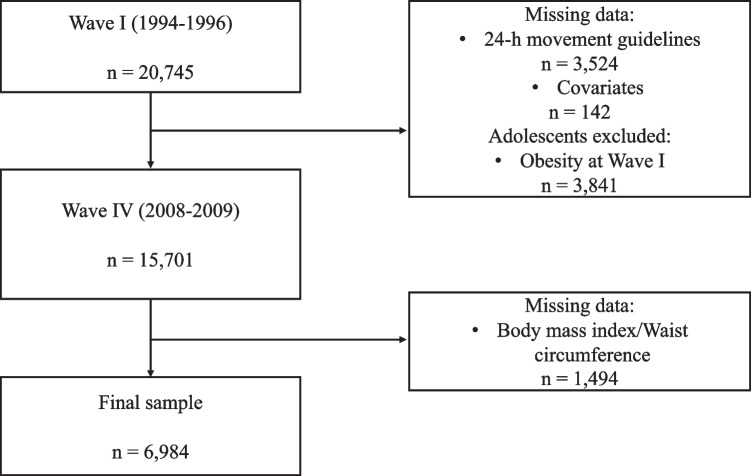
Flow chart of the participants

This is a longitudinal study with data from the Add Health study, a nationally representative sample of adolescents in grades 7–12 in the USA followed from adolescence through adulthood. During 1994 and 1995, over 90,000 students from a sample of US high schools were selected with unequal probability of selection completed in-school questionnaires (i.e., the units in the population do not have the same probability of being included in a sample), and 20,745 of them were selected to participate in the Wave I in-home interview in 1994 [25]. Wave IV in-home sample was followed in 2008–2009 (*n* = 15,701; age range 24–32 years). For this study, we used data from Waves I and IV. Wave I provided data from 24-h movement behaviors. Wave IV provided obesity outcomes (i.e., waist circumference [WC] and body mass index [BMI]). The current analysis included those who completed Wave IV assessment (defined by if they had a Wave IV sample weight; *n* = 15,701). Participants with missing data on 24-h movement behaviors (*n* = 3524), and/or some covariates (*n* = 142) at Wave I, and/or BMI and WC data at Wave IV (*n* = 1494) were excluded. Also, participants with obesity at Wave I (body mass index z-score [zBMI] ≥ 2 standard deviation determined using the International Obesity Task Force growth references chart [26]) (*n* = 3841) were removed. The final sample included 6984 participants (59.4% female) (Fig. 1).

Add Health study was approved by the Institutional Review Board (IRB) at the University of North Carolina at Chapel Hill. The permission to conduct secondary analyses was obtained by the Ethics Committee of the University Hospital of Navarra (PI_2020/143).

Independent samples *t*-test and chi-square analyses were conducted to compare adolescents included in the final sample compared with the remaining participants who were not included in the analysis. There were no differences in any demographic variables (race/ethnicity, *p* = 0.112; region, *p* = 0.151) and parental income (*p* = 0.504) between adolescents who were included or not in the final sample. Therefore, it could be assumed that missing data did not meaningfully influenced results within the analytic sample.

### Procedures

#### Anthropometric measures

At Wave IV, height and weight to the nearest 1/2 pound and height to the nearest 1/8 inch were measured by trained study staff, and BMI was calculated by dividing body weight (in kg) by height (in m [2]). Height and weight at Wave I were self-reported, whereas those at Waves IV were measured by the field examiners following standardized protocols (details can be found in the Add Health website, https://addhealth.cpc.unc.edu/documentation/codebooks/). As above mentioned, adolescents were considered obese when presented a zBMI ≥ to 2 standard deviation using the International Obesity Task Force growth references chart [26]. Adults were classified as participants with obesity if his or her BMI was 30 kg/m [2] or greater [27]. WC was also measured to the nearest 0.5 cm at the superior border of the iliac crest as recommended by the National Cholesterol Education Program Third Adult Treatment Panel during the Wave IV in-home assessment, using a cut-off point of ≥ 102 cm in male and 88 cm in female [28].

#### Twenty-four-hour movement guidelines

In the Wave I in-home interview, adolescents reported their engagement in moderate-to-vigorous physical activity (MVPA) during the past 7 days by a previously described scale and three different questions [29]: “During the past week, how many times did you go rollerblading, roller-skating, skateboarding, or bicycling?”; “During the past week, how many times did you play an active sport, such as baseball, softball, basketball, soccer, swimming, or football?”; “During the past week, how many times did you exercise, such as jogging, walking, karate, jumping rope, gymnastics or dancing?”. Responses ranged from not at all to five or more times and were scored as: 0 times = not at all, 1.5 times = 1 or 2 times, 3.5 times = 3 or 4 times, and 6 times = 5 or more times. Responses to the three questions were summed to create a measure of total times of MVPA each week, classified as no (0 times), some (1–4 times), and high (5 or more times) MVPA per week. Meeting physical activity recommendations was considered when adolescents reported 5 or more times MVPA per week following the Gordon-Larsen et al. [29] criterion.

Screen time was measured by a previously described scale [29], using the following the questions: “How many hours a week do you watch television?” “How many hours a week do you watch videos?” and “How many hours a week do you play video or computer games?” Hours given in the three responses were summed to create a measure of recreational screen time per week. Meeting screen time recommendations was considered when adolescents reported no more than 2 h per day [30].

Adolescents reported their sleep duration (in hours) in response to one question in the Wave I in-home interview: “How many hours of sleep do you usually get per day/night?”. The prevalence of meeting sleep duration guidelines was estimated by the National Sleep Foundation’s sleep duration guidelines from 9 to 11 h and 8 to 10 h per day of sleep [31] (i.e., those adolescents aged 14–17 years who slept less than 8 h and more than 10 h were categorized as “not meeting the guidelines”) in those aged 12–13 and 14–17 years old, respectively.

#### Covariates

Information on sociodemographic factors, such as age, sex, race/ethnicity (operationalized as a four-level: White, Black, native American, and Asian), region (coded as West, Midwest, South, and Northeast), and parental income (range $0 to $999 thousand), was collected through in-home questionnaires.

### Statistical analysis

Descriptive information is shown as numbers and percentages for categorical variables and the mean and standard deviation for continuous variables. Preliminary analyses showed no significant interactions between sex and all three movement behaviors in relation to BMI (*p* = 0.273) and WC (*p* = 0.126) at Wave IV; therefore, all analyses were performed with male and female together. All model assumptions were checked (i.e., normality and homoscedasticity) and both dependent variables were log-transformed (base 10) to normalize the distribution.

We conducted a series of linear regression models to examine the associations between meeting specific and general combinations (i.e., physical activity and screen time, physical activity and sleep duration, screen duration and sleep time, and all three) of 24-h movement guidelines at Wave I with WC and BMI at Wave IV. For these analyses, not meeting the guideline(s) was used as the reference group.

Poisson regression with robust error variance analyses [32] were used to estimate the incidence rate ratio (IRR) of obesity (dependent variable), according to meeting specific and combinations (i.e., physical activity and screen time, physical activity and sleep duration, screen duration and sleep time, and all the three) of 24-h movement guidelines at Wave I (independent variables). In all cases, not meeting the guideline(s) was the reference group. All analyses were adjusted by sex, age, zBMI, race/ethnicity, region, and parental income at baseline. Further, each individual recommendation (i.e., physical activity, screen time, and sleep duration) was additionally adjusted by the rest of behaviors (e.g., physical activity was also adjusted by screen time and sleep duration). We used STATA version 17.0 (StataCorp LLC, College Station, TX, USA) with *SVY* command and set significance at *p* < 0.05. For the analyses, we used population weights so that it is representative of the actual composition of each school based on grade level and gender and corrects for the unequal probability of selection of schools across regions. Finally, we considered the nesting structure of the data in our analyses, by adjusting standard errors proportional to the degree of nesting.

## Results

Descriptive statistics for the full sample at Wave I and at Wave IV are presented in Table 1. At Wave I (age = 15.18 years), only 7.4% of the adolescents met all three recommendations, and 18.7% met none of them. Specifically, 52.3% of the sample reported recommended sleep duration, 40.8% reported recommended screen time use, and 33.2% were physically active. At Wave IV (age = 28.31 years), 21.6% and 40.6% of the sample were classified as participant with obesity according to their BMI and WC, respectively.

**Table 1:** Descriptive characteristics of the analyzed study sample at Wave I

**Waves I (1994)**	***n*= 6984**
Sex (women), *n* (%)	4147 (59.4)
Age, years	15.18 (1.29)
Anthropometric measurements	
Body mass, kg	55.13 (9.36)
Height, m	1.55 (0.10)
Body mass index, kg/m^2^	22.85 (2.71)
Body mass index, z-score	0.97 (0.76)
Race category	
White, n (%)	4889 (70.0)
Black, n (%)	1467 (21.0)
Native American, n (%)	112 (1.6)
Asian, n (%)	519 (7.4)
Region	
West, n (%)	1677 (24.0)
Midwest, n (%)	1767 (25.3)
South, n (%)	2507 (35.9)
Northeast, n (%)	1041 (14.9)
Parental income, $ thousand	1112.74 (3074.79)
Guideline component met	
Physical activity, n (%)	2316 (33.2)
Screen time, n (%)	2850 (40.8)
Sleep duration, n (%)	3656 (52.3)
Physical activity + screen time	930 (13.3)
Physical activity + sleep duration	1262 (18.1)
Screen time + sleep duration	1468 (21.0)
None	1303 (18.7)
All three recommendations	519 (7.4)

Table 2 shows the differences in BMI and WC at follow-up between adolescents that met vs. did not meet the physical activity, screen time, and sleep duration recommendations (individually and combined). Adolescents who met screen time in isolation (β =  − 1.62 cm, 95% CI − 2.68 to − 0.56 cm), jointly with physical activity (β =  − 2.25 cm, 95% CI − 3.75 to − 0.75 cm), and all three recommendations (β =  − 1.92 cm, 95% CI − 3.81 to − 0.02 cm) obtained lower WC at Wave IV than those who did not meet any of these recommendations.

**Table 2:** Differences in BMI and WC at follow-up between adolescents that met vs. not met physical activity, screen time, and sleep duration recommendations and combinations of these recommendations

	Body mass index (kg/m^2^)		Waist circumference (cm)	
	β (95% CI)	*p*	β (95% CI)	*p*
Meeting (vs. not meeting) individual guideline				
Physical activity^a^	− 0.30 (− 0.73 to 1.28)	0.169	− 0.90 (− 1.95 to 0.14)	0.091
Screen time^a^	− 0.27 (− 0.71 to 1.65)	0.223	**− 1.62 (− 2.68 to -0.56)**	**0.003**
Sleep duration^a^	0.32 (− 0.11 to 0.78)	0.145	0.84 (− 0.20 to 1.89)	0.112
Meeting (vs. not meeting) specific combinations				
Physical activity & Screen time	− 0.48 (− 1.12 to 0.15)	0.133	**− 2.25 (− 3.75 to − 0.75)**	**0.003**
Physical activity & Sleep duration	− 0.16 (− 0.64 to 0.31)	0.499	− 0.03 (− 1.19 to 1.12)	0.951
Screen time & Sleep duration	− 0.09 (− 0.58 to 0.40)	0.724	− 1.08 (− 2.27 to 0.12)	0.077
All three recommendations	− 0.31 (− 1.09 to 0.47)	0.436	** − 1.92 (− 3.81 to − 0.02)**	**0.048**

Analysis adjusted by sex, age, zBMI, race/ethnicity, region, and parental income at baseline

to aid interpretation, data were back-transformed from the log scale for presentation in the results

*CI* confidence interval

^a^Each independent individual guideline was additionally adjusted by the rest of behaviors (e.g., physical activity was also adjusted by screen time and sleep duration)

Finally, Table 3 shows the IRR for obesity associated with meeting vs. not meeting 24-h movement guidelines. Adolescents who only met screen time recommendations (IRR = 0.84, 95% CI 0.76 to 0.92), adolescents who met screen time jointly with physical activity recommendations (IRR = 0.86, 95% CI 0.67 to 0.97), and adolescents who met all the three recommendations (IRR = 0.76, 95% CI 0.60 to 0.97) had lower odds of abdominal obesity at adulthood than those who did not meet any of these recommendations.

**Table 3:** Incidence rate ratio for obesity at Wave IV associated with meeting vs. not meeting physical activity, screen time, and sleep duration and combinations of these recommendations during adolescence

	Obesity		Abdominal obesity	
Meeting the following recommendation	IRR (95% CI)	*p*	IRR (95% CI)	*p*
Meeting (vs. not meeting) individual guideline				
Physical activity^a^	0.93 (0.78 to 1.10)	0.406	0.97 (0.87 to 1.08)	0.588
Screen time^a^	0.94 (0.80 to 1.09)	0.418	**0.84 (0.76 to 0.92)**	**< 0.001**
Sleep duration^a^	1.18 (0.94 to 1.37)	0.06	1.02 (0.93 to 1.12)	0.683
Meeting (vs. not meeting) specific combinations				
Physical activity and Screen time	0.86 (0.67 to 1.10)	0.242	**0.86 (0.67 to 0.97)**	**0.042**
Physical activity and Sleep duration	0.97 (0.79 to 1.18)	0.755	0.97 (0.79 to 1.18)	0.755
Screen time and Sleep duration	0.96 (0.80 to 1.15)	0.661	0.96 (0.80 to 1.15)	0.661
All three recommendations	0.96 (0.67 to 1.38)	0.85	**0.76 (0.60 to 0.97)**	**0.027**

Analysis adjusted by sex, age, zBMI, race/ethnicity, region, and parental income at baseline

*IRR* incidence rate ratio

^a^Each independent variable was additionally adjusted by the rest of behaviors (e.g., physical activity was also adjusted by screen time and sleep duration)

## Discussion

To our knowledge, this is the first study that determined the relationship between meeting the 24-h movement guidelines from adolescence with obesity at adulthood 14 years later. Overall, our results show that meeting screen time alone or jointly with physical activity recommendations during adolescence was linked to lower WC and abdominal obesity at adulthood. In addition, adolescents who met all movement behavior recommendations had lower risk of presenting abdominal obesity later in life.

A growing body of evidence highlights the health advantages of increasing physical activity and reducing sedentary behaviors for all populations [33]. Despite this, the prevalence of insufficient physical activity remains a worrying public health concern in both adolescents [34] and adults [35]. Scientific literature also shows that young population in most of the countries present low prevalence of overall physical activity levels [36], a high prevalence of sedentary behavior levels [36], and an increasing prevalence of obesity [37]. The long-term results of our study confirm and support previously reported findings of an earlier study that analyzed the same cohort (wave III), which found that adolescent screen time, but not physical activity was related with incidence of general body obesity at 21 years old [38]. Our results continue to show this association, but with a longer follow-up; seven years later. Also, our results show that meeting both physical activity and screen time recommendations jointly during adolescence was associated with lower abdominal obesity at adulthood 14 years later. Supporting our results, one systematic review suggested that increased physical activity and decreased sedentary behavior are protective against relative weight and fatness gains over childhood and adolescence [39]. Similarly, some studies using isotemporal substitution models reported that reallocating time from sedentary behavior to moderate-to-vigorous physical activity showed significant reductions in several obesity indicators, such as WC [40, 41], body fat percentage [42] or BMI [41]. Further, a longitudinal study by Barbour-Tuck et al. [43] showed the importance of engaging in high levels of physical activity to mitigate the accumulation of fat mass in the trunk and prevent the transition from having healthy weight to excess weight during emerging adulthood.

In relation to sleep duration, a longitudinal study by Sokol et al. [44] suggested that greater BMI could lead shorter sleep during adolescence to young adulthood. Similarly, one meta-analysis by Fatima et al. [45] provided evidence that short sleep duration in young subjects is significantly associated with future overweight/obesity. Despite this fact, we were not able to confirm this association in our study when we assessed sleep duration alone; not being so when it was analyzed in combination with the rest of movement behaviors. This could be because movement behaviors could have accumulative effects on health when considered together rather than individually [46].

In line with the above, it is noteworthy that, until recently, studies determining the influence of physical activity, sedentary behavior, and sleep duration on different health outcomes have mainly been conducted individually or separately from the other behaviors [6]. Notwithstanding, since time is limited (i.e., 24-h), adolescents have to make choices between different activities during the day, so it is conceptually wrong to consider that the impact of a particular behavior is independent of other [47]. In this sense, when we analyzed all behaviors together, we observed that meeting all behaviors recommendations during adolescence was linked to lower odds of presenting abdominal obesity during adulthood, but not with general body obesity. For young population, scientific literature shows strong evidence about the associations between adherence to all three 24-h movement guidelines and reduced body fat mass and lower risk of having obesity [6]. Furthermore, a longitudinal study by Chemtob et al. [22] that evaluated the relationship between adiposity (from childhood to adolescence) and 24-h movement guidelines showed greater body fat mass 2 and 7 years later in those who did not meet the guidelines [22]. Another longitudinal study carried out by Micklesfield et al. [23] found less optimal body composition (in girls) in those who were constantly physically active, had a higher sleep duration, and were more sedentary through adolescence, in their study conducted in South Africa [23]. There are some possible explanations that could justify our findings. Firstly, movement behaviors are codependent since one movement behavior can replace an equal amount of time of one (or more) of the others [46] (e.g., sedentary behaviors that displace physical activity). Supporting this fact, a study by Kim et al. [48] with a composited data analysis showed reductions in BMI when replacing sedentary behaviors by physical activity or sleep time. Moreover, it has been pointed out that changes from a physically active to a more sedentary lifestyle in later life entails a reduction of energy consumption [49]. Secondly, physical activity has a dose–response relationship, is time-consistent and has biological plausibility on obesity development and, also, the reduction in physical activity levels is considered a key factor in the increasing worldwide prevalence of obesity [50]. Also, there are a number of studies which have specifically investigated the effect of physical activity on abdominal obesity, irrespective of total body weight [51]. For example, Lee et al. [52] found that, even in the absence of weight loss, moderate-intensity exercise (30 – 60 min of brisk walking) was associated with significant reductions in total, abdominal fat, and WC. This could be one of the possible reasons which could explain the lack of association with general body obesity in our study. Finally, short sleep duration has shown to significantly increase the risk of obesity [16, 45], due to some suggested factors, such as endocrine and metabolic changes, increased appetite which could provoke a greater caloric intake, higher systemic inflammation and reduced physical activity linked to daytime sleepiness [53].

There are several limitations that should be declared. Firstly, the longitudinal study design does not allow us to establish causal-effect relationships. Secondly, information about movement behaviors were self-reported by adolescents, which is subject to bias. A stronger methodology would be more appropriate to obtain objective measures of physical activity, sedentary behavior and sleep duration (e.g., accelerometers). Also, we classified respondents as meeting physical activity recommendations when they performed MVPA five or more times per week. The physical activity instrument used is limited in scope since does not include a school component and give no indication of time, which makes it really difficult to pinpoint if youth met the 60 min of MVPA per day recommendation. Therefore, results need to be interpreted with caution. Thirdly, we did not include information about dietary patterns, which could influence on obesity markers as previously suggested [54]. Moreover, dataset from 1994 to 1996 is being used to gauge meeting a recommendation made later. Given that the prevalence and conditions in which sedentary behaviors occur have changed in the last two decades, findings of this study should be interpreted with caution. Also, screen time questions did not specifically mention “leisure screen-based activities” and therefore we cannot be certain that this includes only “recreational screen time”. Lastly, height and weight at Wave I were self-reported. However, self-reported height and weight in the Add Health cohort seems to be highly correlated with measured height and weight at later waves [55].

In conclusion, our results show that meeting screen time separately, jointly with physical activity recommendations, and meeting all movement behavior recommendations during adolescence was associated with lower risk of abdominal obesity 14 years later in a nationally representative US cohort, therefore, our findings may not be generalizable outside the USA. Our study highlights the importance of promoting the adherence to the 24-h movement guidelines during adolescence to prevent the risk of suffering abdominal obesity later in life. Nevertheless, further studies with robust measurements are required to confirm our findings.

## Data Availability

The data and materials are available and can be provided by the corresponding author, if based upon reasonable request and based on a study protocol.
